# Wnt Isoform-Specific Interactions with Coreceptor Specify Inhibition or Potentiation of Signaling by LRP6 Antibodies

**DOI:** 10.1371/journal.pone.0012682

**Published:** 2010-09-13

**Authors:** Yan Gong, Eric Bourhis, Cecilia Chiu, Scott Stawicki, Venita I. DeAlmeida, Bob Y. Liu, Khanhky Phamluong, Tim C. Cao, Richard A. D. Carano, James A. Ernst, Mark Solloway, Bonnee Rubinfeld, Rami N. Hannoush, Yan Wu, Paul Polakis, Mike Costa

**Affiliations:** 1 Department of Cancer Targets, Genentech Research & Early Development, South San Francisco, California, United States of America; 2 Department of Protein Engineering, Genentech Research & Early Development, South San Francisco, California, United States of America; 3 Department of Antibody Engineering, Genentech Research & Early Development, South San Francisco, California, United States of America; 4 Department of Molecular Biology, Genentech Research & Early Development, South San Francisco, California, United States of America; 5 Department of Tumor Biology and Angiogenesis, Genentech Research & Early Development, South San Francisco, California, United States of America; 6 Department of Protein Chemistry, Genentech Research & Early Development, South San Francisco, California, United States of America; Max Planck Institute of Molecular Cell Biology and Genetics, Germany

## Abstract

β-catenin-dependent Wnt signaling is initiated as Wnt binds to both the receptor FZD and coreceptor LRP5/6, which then assembles a multimeric complex at the cytoplasmic membrane face to recruit and inactivate the kinase GSK3. The large number and sequence diversity of Wnt isoforms suggest the possibility of domain-specific ligand-coreceptor interactions, and distinct binding sites on LRP6 for Wnt3a and Wnt9b have recently been identified *in vitro*. Whether mechanistically different interactions between Wnts and coreceptors might mediate signaling remains to be determined. It is also not clear whether coreceptor homodimerization induced extracellularly can activate Wnt signaling, as is the case for receptor tyrosine kinases. We generated monoclonal antibodies against LRP6 with the unexpected ability to inhibit signaling by some Wnt isoforms and potentiate signaling by other isoforms. In cell culture, two antibodies characterized further show reciprocal activities on most Wnts, with one antibody antagonizing and the other potentiating. We demonstrate that these antibodies bind to different regions of LRP6 protein, and inhibition of signaling results from blocking Wnt binding. Antibody-mediated dimerization of LRP6 can potentiate signaling only when a Wnt isoform is also able to bind the complex, presumably recruiting FZD. Endogenous autocrine Wnt signaling in different tumor cell lines can be either antagonized or enhanced by the LRP6 antibodies, indicating expression of different Wnt isoforms. As anticipated from the roles of Wnt signaling in cancer and bone development, antibody activities can also be observed in mice for inhibition of tumor growth and in organ culture for enhancement of bone mineral density. Collectively, our results indicate that separate binding sites for different subsets of Wnt isoforms determine the inhibition or potentiation of signaling conferred by LRP6 antibodies. This complexity of coreceptor-ligand interactions may allow for differential regulation of signaling by Wnt isoforms during development, and can be exploited with antibodies to differentially manipulate Wnt signaling in specific tissues or disease states.

## Introduction

Similar to most other morphogen and growth factor signaling pathways, mammalian Wnt signaling is deployed multiple times during development and tissue homeostasis [1]. These events differentially utilize 19 ligands, 10 receptors, and multiple coreceptors, including LRP5/6, Ror1/2, and Ryk [2]. In addition, different secreted antagonists that bind either Wnts, such as SFRP1/2/3/4/5 and WIF1, or bind LRP5/6, including DKK1/2/4 and SOST, modulate interactions between ligands and receptors. These membrane and extracellular proteins and their multiple isoforms provide for differential regulation at the level of expression and combinatorial protein interactions. Most Wnt isoforms appear to be capable of binding coreceptor LRP5/6, and this engagement specifies canonical, or β-catenin dependent, Wnt signaling. Wnt-mediated heterodimerization of LRP5/6 and FZD triggers phosphorylation of the LRP5/6 intracellular domain and Axin binding [3]–[5]. DVL is brought into the complex by directly binding both Axin and FZD, and DVL oligomerization likely enlarges these protein complexes on the cytoplasmic face of the membrane to sequester GSK3 and inhibit its phosphorylation and destabilization of β-catenin [6]–[12].

The unusually large number of ligand isoforms that mediate mammalian canonical Wnt signaling display considerable primary sequence divergence. This diversity contrasts with the pair of highly homologous coreceptors. The LRP5 and LRP6 extracellular domains consist largely of four homologous regions, named E1 to E4 from N- to C-terminal, each containing a YWTD-type β-propeller and EGF-like domain [13]. Each repeat at a similar position in LRP5 and LRP6 is highly conserved, whereas the different repeats within the same protein are considerably more divergent. Interestingly, Bourhis *et al*. (2010) demonstrated that Wnt9b binds exclusively within the E1-E2 region *in vitro*, whereas Wnt3a binds only to a fragment containing E3-E4, suggesting that each repeat, or a combination of two adjacent repeats, binds to a different subset of Wnt isoforms [14]. This arrangement may accommodate the diversity of Wnt proteins, and possibly also allow for their differential regulation by LRP5/6 antagonist ligands. By contrast, in Notch and VEGF receptors, whose extracellular regions contain repeats of EGF-like and Ig domains, respectively, binding of multiple ligand isoforms is localized to the same region of one or two repeats, although the presence of other repeats can enhance binding [15]–[17].

For receptor tyrosine kinases, ligand-induced dimerization stimulates the kinase activity and initiates signaling. While ligand-induced receptor-coreceptor heterodimerization is necessary for canonical Wnt signaling, there is no clearly defined role for LRP5/6 or FZD homodimerization. Forced dimerization of different recombinant LRP6 proteins can either activate or inhibit Wnt signaling [18], [19]. In this report, we utilize monoclonal antibodies to further explore the functional roles of different regions of LRP6 in signaling by Wnt isoforms and in antagonism by DKK1, as well as the functional consequences of dimerizing endogenous coreceptor. We provide evidence that different subsets of Wnt proteins induce signaling through binding different regions of LRP6, with most Wnts interacting within either E1-E2 or E3-E4, but not both. Individual LRP6 antibodies can inhibit autocrine, or endogenous, Wnt signaling in some tumor cell lines and potentiate autocrine signaling in other tumor cells, likely because the cell lines express different Wnt isoforms. Antibody-mediated dimerization of LRP6 does not induce signaling by itself, but it does potently enhance, or potentiate, signaling in the presence of a Wnt isoform that can simultaneously bind LRP6 and presumably recruit FZD into the complex. With increased understanding of the different Wnt isoforms mediating proliferation, cell fate specification, and stem cell self-renewal in different cancer types and developmental processes, antibodies targeting different regions of LRP6 may prove to be effective agents for selectively modulating these processes.

## Results

### Isolation of Wnt Antagonist and Potentiating LRP6 Monoclonal Antibodies

**Figure 1:**
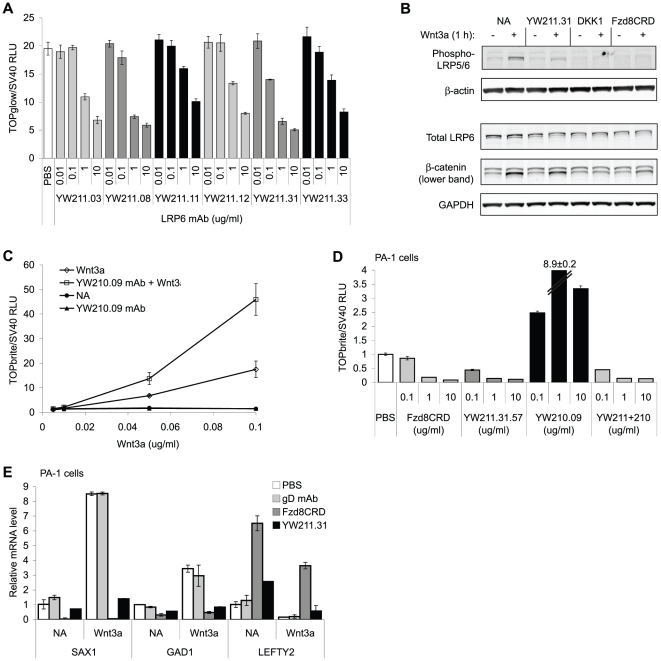
LRP6 monoclonal antibodies antagonize and potentiate Wnt3a-induced signaling and teratocarcinoma autocrine Wnt signaling. (A) Six antibodies against LRP6 E3-E4 protein fragment inhibit in a concentration-dependent manner the Wnt luciferase reporter activity in HEK293 cells induced with 0.1 µg/ml purified Wnt3a. Error bars of this and all other graphs, except where noted, represent standard deviation of at least 3 replicate samples. (B) Western analysis of HEK293 cells either unstimulated (−) or induced (+) with Wnt3a and treated with the indicated LRP6 antibody or purified protein. RNAi experiments demonstrate that only the lower molecular weight band recognized by the β-catenin polyclonal antibody represents β-catenin protein (data not shown). β-actin and GAPDH protein levels are shown as sample loading controls for the upper and lower gels, respectively. (C) YW210.09 antibody potentiates Wnt reporter gene activity in a manner proportional to Wnt3a concentration in HEK293 cells (NA indicates no addition of Wnt3a or antibody). (D) Concentration-dependent inhibition and potentiation of autocrine Wnt signaling in PA-1 teratocarcinoma cells transfected with luciferase reporters and treated with LRP6 antibodies, either individually or in combination, or with Fzd8CRD-Fc protein. (E) qPCR expression analysis of Wnt-induced genes SAX1 and GAD1 and Wnt-repressed gene LEFTY2 in PA-1 cells treated with or without (NA) 0.3 µg/ml Wnt3a protein, and treated with 10 µg/ml YW211.31 antibody, anti-gD monoclonal antibody as a negative control, or Fzd8CRD-Fc protein as a positive control.

To develop candidate therapeutic molecules to manipulate Wnt signaling, we generated antibodies that can either inhibit or enhance signaling induced by Wnt3a protein. We used recombinant LRP6 E1-E2-Fc and E3-E4-Fc proteins to select from human synthetic antibody phage libraries and confirmed binding of isolated phage clones to LRP6 by ELISA and FACS [20]. Twenty-four unique antibody heavy chain clones against LRP6 E1-E2 and 22 unique clones against E3-E4 were reformatted and expressed as full-length human IgG1 antibodies. In luciferase reporter gene assays, six of the LRP6 E3-4 (Figure 1A) and none of the E1-E2 antibodies (data not shown) inhibited Wnt3a-induced signaling in HEK293 cells. The YW211.31 antibody recognizing the LRP6 E3-4 domain is the most potent in inhibiting signaling in Wnt3a-stimulated HEK293 cells, with an IC_50_ of approximately 1 µg/ml (or 6 nM). YW211.31 antibody inhibits Wnt3a-induced LRP6 phosphorylation and β-catenin protein stabilization without affecting levels of LRP6 protein, similar to purified Wnt-binding protein Fzd8CRD-Fc [21] and LRP6-binding protein DKK1 (Figure 1B). YW211.31 antibody can also antagonize mouse LRP6 function since it partially inhibits Wnt3a-induced reporter activity in mouse NIH/3T3 cells and β-catenin protein stabilization in mouse L cells (data not shown).

YW211.31 antibody has a binding affinity of about 2 nM by surface plasmon resonance (SPR) and 0.6 nM by Scatchard analysis. To improve affinity and potential potency of YW211.31 antibody, the clone was affinity-matured using His-tagged LRP6 E3-E4 protein and CDR combinatorial libraries in which selected CDR residues were targeted for randomization. Four phage clones showing the most improved affinity by phage competition ELISA were reformatted and expressed as full length human IgGs. The dissociation rate constants of all four affinity-matured IgGs were decreased, leading to improved affinities for the best two antibodies, YW211.31.57 and YW211.31.62, of K_D_ 0.27 and 0.17 nM, respectively. YW211.31.57 and YW211.31.62 also show improved potency in inhibiting signaling in Wnt3a-stimulated HEK293 cells, with IC_50_ values of approximately 0.1 µg/ml (0.6 nM).

None of the antibodies isolated in the screen activated signaling in HEK293 cells in the absence of stimulation with exogenous Wnt3a protein, however five of the LRP6 E1-E2 and two of the E3-E4 antibodies potentiated Wnt3a-induced signaling at least 2-fold. In mouse NIH/3T3 cells, YW210.09, an E1-E2 antibody, also potentiated Wnt3a-induced signaling at least 1.5-fold, indicating that it also recognizes mouse LRP6 (data not shown). In HEK293 cells, the magnitude of enhancement of Wnt3a-induced signaling by YW210.09 antibody is proportional to Wnt3a concentration (Figure 1C). YW210.09 antibody interacts with human LRP6 E1-E2 protein with a K_D_ of 5 nm as measured by SPR analysis. ELISA testing shows that all antagonist and potentiating antibodies specifically bind only the LRP6 protein fragment employed for their isolation, and none recognize both E1-E2 and E3-E4. FACS analysis indicates that soluble LRP6 E1-E4 protein efficiently and completely blocks binding of YW211.31.57 and YW210.09 to HEK293 cells, suggesting that these antibodies do not recognize other cell surface proteins (Figure S1).

### Effects of LRP6 Monoclonal Antibodies on Autocrine Wnt Signaling

We next determined whether the LRP6 antibodies could antagonize or potentiate endogenous, or autocrine, Wnt signaling detected in a variety of tumor cell lines [21]–[23]. In teratocarcinoma cell lines PA-1 and NTERA-2, YW211.31 antibody inhibits reporter activity induced by autocrine Wnt signaling (Figure 1D) with similar potency to that observed with exogenous Wnt3a (data not shown). In PA1 cells, inhibition of Wnt signaling by YW211.31 antibody is also observed for expression of endogenous Wnt target genes (Figure 1E). The antibody partially inhibits expression of SAX1, GAD1, and APCDD1 that is either induced by exogenous Wnt3a protein or maintained by endogenous, autocrine Wnt signaling. Conversely, repression of LEFTY2 expression by either Wnt3a protein or autocrine Wnt signaling is relieved by YW211.31 antibody. In contrast, YW210.09 antibody potentiates both autocrine Wnt signaling (Figure 1D) and Wnt3a-induced signaling (data not shown) in PA-1 cells by reporter gene assays. Whereas inhibition of Wnt signaling by YW211.31.57 antibody increases progressively with greater antibody concentration, potentiation by YW210.09 and other antibodies decreases at high antibody concentrations in some cell types, such as PA-1 cells. This may suggest that antibody-mediated LRP6 dimerization is required for potentiation, since high antibody concentrations would favor monovalent interactions and thus limit crosslinking of LRP6 molecules. Treatment of PA-1 cells with a combination of both YW211.31.57 and YW210.09 antibodies antagonizes both Wnt3a-induced and autocrine Wnt signaling, similar to the effect of YW211.31.57 alone (Figure 1D).

**Figure 2:**
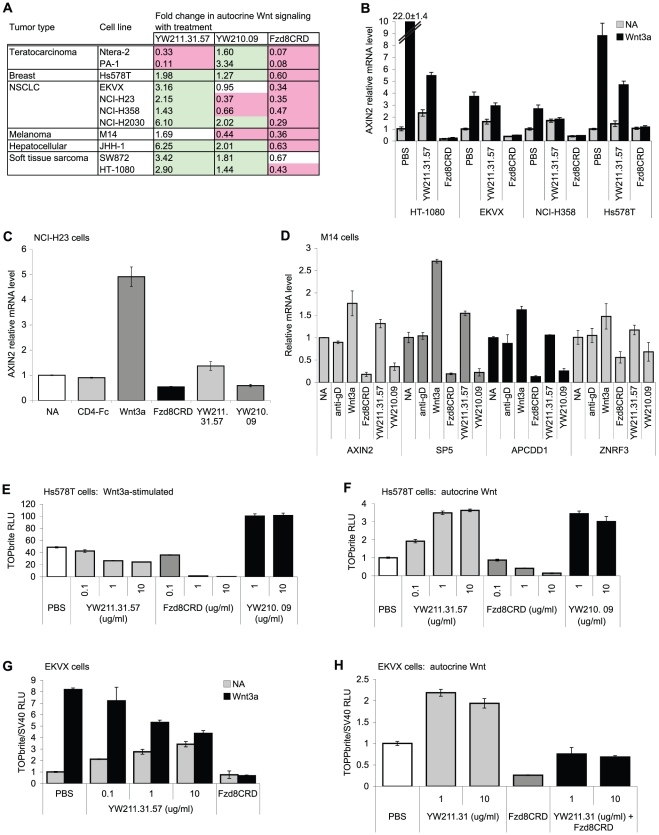
LRP6 antibodies can have different effects on autocrine Wnt signaling in other cell lines. (A) Summary table of 11 cell lines displaying autocrine Wnt signaling inhibited by Fzd8CRD-Fc protein (10 µg/ml), and the effects of LRP6 antibodies on autocrine signaling. The fold change in autocrine Wnt signaling is highlighted in red or green to indicate inhibition or potentiation, respectively, with p-value <0.01 by one-way analysis of variance (ANOVA). Antibodies were used at 10 µg/ml, except for NCI-H358 and HT-1080 cells which were treated with 1 µg/ml YW211.31.57 or YW210.09 antibody, respectively, to increase potentiation effects. (B) qPCR expression analysis of AXIN2 mRNA in four cell lines treated with 25 µg/ml YW211.31.57 antibody or Fzd8CRD-Fc protein, with and without (NA) 0.2 µg/ml Wnt3a. (C–D) Expression of Wnt-induced genes in NCI-H23 (C) and M14 (D) cells is potentiated by YW211.31.57 and antagonized by YW210.09 antibody (30 µg/ml). CD4-Fc protein (30 µg/ml) serves as a negative controls (C). For M14 cells (D), AXIN2 and SP5 expression is potentiated more potently by Wnt3a protein or YW211.31.57 antibody than is APCDD1 and ZNRF3 expression. (E–F) In Hs578T cells stably integrated with Wnt luciferase reporter, YW211.31.57 antibody shows concentration-dependent inhibition of Wnt3a-stimulated signaling (E) and potentiation of autocrine Wnt signaling (F). Fzd8CRD-Fc protein inhibits and YW210.09 antibody potentiates signaling with or without 0.1 µg/ml Wnt3a stimulation. RNAi experiments indicate that at least 41% of Wnt3a-induced signaling in Hs578T cells is dependent on LPR5 expression (data not shown), and this signaling is predicted to be inhibited by Fzd8CRD-Fc protein but not YW211.31.57 antibody. In this experiment, SV40-driven luciferase was not transfected for normalization and, instead, antibody and protein treatments were independently confirmed to have no significant effect on viability of this cell line. (G–H) EKVX cells transfected with Wnt luciferase reporter also display potentiation of autocrine Wnt signaling (NA) and antagonism of Wnt3a-induced signaling by YW211.31.57 antibody. Antibody-mediated potentiation of autocrine Wnt signaling is inhibited by 5 µg/ml Fzd8CRD-Fc protein (H).

To identify additional cell lines that display autocrine Wnt signaling, we tested cell lines with relatively high expression of Axin2 mRNA or phospho-LRP5/6 protein for inhibition of Wnt signaling by Fzd8CRD-Fc protein in Wnt luciferase reporter gene assays. Nine cell lines exhibited autocrine Wnt signaling that was inhibited by Fzd8CRD-Fc protein (Figure 2A), including NSCLC cells NCI-H23 and NCI-H2030 and soft tissue sarcoma cells SW872 and HT-1080 that have previously been reported to have endogenous Wnt signaling based on assays with other Wnt antagonists [23]–[25]. When signaling was further induced in all nine cell lines by exogenous Wnt3a protein, YW211.31.57 antibody inhibited this response to Wnt3a (Figure 2A, 2B, 2E, and 2G). Surprisingly, YW211.31.57 antibody potentiated autocrine Wnt signaling in all nine of these cell lines, whereas YW210.09 potentiated autocrine Wnt signaling in five lines and inhibited in three lines (Figure 2A–2D, 2F, and 2G). This reciprocal activity of YW211.31.57 antibody on autocrine and Wnt3a-induced signaling was observed not only using the luciferase reporter, but also for expression of endogenous Wnt target genes such as Axin2 in the six cell lines tested (Figure 2B, 2C, and 2D). In EKVX (Figure 2H) and breast carcinoma Hs578T (data not shown) cell lines, we confirmed that the increase in Wnt signaling by YW211.31 antibody was dependent on autocrine Wnt(s) by demonstrating that this increase is blocked by Fzd8CRD-Fc protein. Potentiation of autocrine Wnt signaling in EKVX and Hs578T cells is also observed with the other five antibody antagonists of Wnt3a-induced signaling identified in the screen (data not shown).

### Reciprocal Activities of LRP6 Antibodies on Different Wnt Isoforms

**Figure 3:**
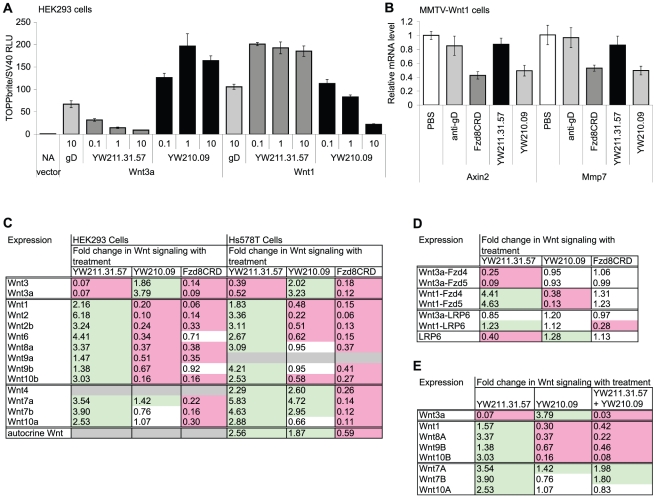
Wnt isoforms specify inhibitory or potentiating activities of LRP6 antibodies. (A) YW211.31.57 antibody antagonizes Wnt3a-induced signaling and potentiates Wnt1-induced signaling, whereas YW210.09 antibody exhibits reciprocal activities. HEK293 cells stably integrated with Wnt and SV40 luciferase reporters were transfected with Wnt3a, Wnt1, or empty vector expression constructs and treated with monoclonal antibodies (mAb). (B) Treatment of cultured cells from mouse MMTV-Wnt1 allograft tumors with 10 µg/ml YW210.09 antibody inhibits expression of Wnt target genes Axin2 and Mmp7, as does 10 µg/ml Fzd8CRD-Fc protein. (C–E) Summary tables of the effects of 10 µg/ml LRP6 antibodies, antibody combinations (E), and Fzd8CRD-Fc protein on signaling induced by transfection of expression constructs for Wnt isoforms (C, E) or chimeric proteins fusing Wnt isoforms to FZD isoforms or LRP6 (D) in HEK293 (C–E) or Hs578T (C) cell lines stably integrated with Wnt luciferase reporters. Expression of Wnt luciferase reporter is normalized to cell number and additionally normalized to levels in cells transfected with the same expression construct but not treated with proteins. The fold change in Wnt signaling is highlighted in red or green to indicate inhibition or potentiation, respectively, with p-value <0.01 by ANOVA. Gray fields indicate conditions that were not tested because Wnt induction was not significant.

Our results show that YW211.31 antibody inhibits signaling induced by exogenous Wnt3a protein in all cell lines, but can either inhibit or potentiate autocrine Wnt signaling in a cell line-dependent manner. This suggests that the specific Wnt isoform driving the autocrine signal dictates the activity of the antibody. Therefore, we tested the activity of the antibody on signaling induced by exogenous expression of Wnt3a and other Wnt isoforms. Wnt signaling induced by transfection of Wnt3a in either HEK293 (Figure 3A) or Hs578T (data not shown) cells is inhibited by YW211.31.57 antibody with similar potency to inhibition of signaling induced by Wnt3a protein treatment. Importantly, we found that signaling induced by Wnt1 expression in both cell lines was potentiated by YW211.31.57 antibody, whereas both Wnt1 and Wnt3a signaling are inhibited by Fzd8CRD-Fc protein as expected. Potentiation of Wnt1 signaling was also observed with the other Wnt3a antagonist antibodies identified in the screen (data not shown). YW210.09 antibody also displayed opposing activities against Wnt3a- and Wnt1-induced signaling, which were the reciprocal of YW211.31.57 activities; *i.e.*, potentiation of Wnt3a and inhibition of Wnt1 signaling (Figure 3A). YW210.09 antibody also inhibits endogenous Wnt1 signaling in cultured tumor cells grown from MMTV-Wnt1 tumors, as observed by reduced expression of Wnt target genes Axin2 and Mmp7 to a similar extent as Fzd8CRD-Fc protein treatment (Figure 3B). In MMTV-Wnt1 cells, YW211.31.57 antibody failed to potentiate Wnt1 signaling, possibly because Wnt1 signaling is already maximal in these cells.

Having demonstrated that YW211.31.57 and YW210.09 antibodies have reciprocal activities on Wnt signaling initiated by Wnt3a and Wnt1, we extended this analysis to the additional 11 of 19 Wnt genes that induce the luciferase reporter greater than two-fold in HEK293 cells (Figure 3C). Only Wnt3a and Wnt3 activities are inhibited by YW211.31.57 antibody, and both are potentiated by YW210.09. Seven Wnt isoforms in addition to Wnt1 are potentiated by YW211.31.57 and inhibited by YW210.09. A third class of Wnt isoforms (Wnt7a, 7b, and 10a) exhibit signaling activity that is not inhibited by either antibody but is potentiated by at least YW211.31.57. In Hs578T cells transfected with different Wnt isoforms, the antibodies display most of these same activities. In particular, YW211.31.57 inhibits Wnt3a and Wnt3 and potenitates all 11 of the other Wnt isoforms that induce the luciferase reporter at least two-fold. YW210.09 also potentiates Wnt3 and Wnt3a in Hs578T cells, as well as inhibits 5 of the 7 Wnt isoforms that are antagonized in HEK293 cells and can be tested in Hs578T cells. The other two Wnt isofoms in this class, Wnt8a and Wnt9b, are not significantly affected by YW210.09 antibody in Hs578T cells. Since RNAi experiments indicate that Wnt3a signaling is transduced by both LRP6 and LRP5 in Hs578T cells, but only by LPR6 in HEK293 cells (data not shown), Wnt8a and Wnt9b might signal primarily through LRP5 in Hs578T cells. Similar to HEK293 cells, Wnt isoforms in the third class are not inhibited by either antibody in Hs587T cells, and we can add to this class Wnt4, which did not induce signaling in HEK293 cells. Only the activity of Wnt7b in this class behaves differently in that YW210.09 antibody potentiates its signaling in Hs578T but not HEK293 cells. In contrast to the Wnt isoform-specific activities of YW211.31.57 and YW210.09 antibodies, Fzd8CRD-Fc protein can potently inhibit the activity of all Wnts tested except Wnt6 and Wnt9b in HEK293 cells.

In Hs578T cells, YW211.31.57 and YW210.09 antibodies potentiate both autocrine Wnt signaling and signaling induced by expression of Wnt4, Wnt7a, and Wnt7b. Thus these three Wnt isoforms are candidates for those that drive autocrine signaling in Hs578T cells. Multiple siRNAs against Wnt7b, but not the other Wnt isoforms, inhibit autocrine signaling in Hs578T cells, identifying the specific Wnt protein mediating signaling (Figure S2C). Since autocrine Wnt signaling in PA-1 cells is inhibited by YW211.31.57 antibody and potentiated by YW210.09, Wnt3 or Wnt3a likely activate endogenous signaling in these cells. Indeed, siRNAs against Wnt3 but not Wnt3a inhibit autocrine Wnt signaling in PA-1 cells (Figure S2A). In NCI-H23 NSCLC cells and in M14 melanoma cells, potentiation of autocrine Wnt signaling by YW211.31 antibody and antagonism by YW210.09 are consistent with Wnt2 RNAi inhibiting endogenous signaling in NCI-H23 cells [23], and with endogenous Wnt1 expression in M14 cells [35]. Using multiple siRNAs, we confirm that Wnt2 expression in NCI-H23 cells (Figure S2D) and Wnt1 in M14 cells (Figure S2B) are required for autocrine Wnt signaling.

### Wnt Isoforms Specify Different Activities of LRP6 Antibodies

Different Wnt isoforms may preferentially bind different FZD isoforms expressed endogenously in the various cell lines, and could conceivably account for the differential activities of our LRP6 antibodies. To examine this possibility, we expressed chimeric proteins covalently linking different Wnt-FZD pairs to test whether the specific Wnt or FZD isoform determines the activity of the LRP6 antibody. Wnt3a or Wnt1 fused to either FZD4 or FZD5 potently activates Wnt signaling in HEK293 cells in the presumed absence of endogenous Wnt expression, whereas overexpression of FZD4 or FZD5 does not induce Wnt signaling (data not shown). YW211.31.57 antibody inhibits the signaling activity of Wnt3a fused to either FZD4 or FZD5, and potentiates the activity of Wnt1 fused to either FZD4 or FZD5 (Figure 3D). YW210.09 antibody shows the reciprocal activity against the Wnt1 chimeras, inhibiting both. Thus the activity of the antibody correlates with the Wnt isoform, and not the FZD isoform. Fzd8CRD-Fc protein has no effect on signaling induced by any of the four Wnt-FZD chimeras, consistent with the chimeras functioning independently of the FZD-binding site of Wnts.

Expression of chimeras that fuse Wnt1 or Wnt3a to LRP6 induce Wnt signaling much more potently than LRP6 overexpression (data not shown). YW211.31.57 and YW210.09 antibodies are not able to inhibit this induction, consistent with the hypothesis that the inhibitory function of the antibodies is dependent on blocking Wnt binding to LRP6 (Figure 3D). It is not clear why the activity of the Wnt-LRP6 chimeras are also largely insensitive to potentiation of Wnt signaling by the antibodies, nor why the Wnt3a-LRP6 fusion is insensitive to Fzd8CRD-Fc inhibition. The lower level of signaling induced by LRP6 overexpression is weakly affected by YW211.31.57 and YW210.09 antibodies, suggesting that a normally undetectable level of endogenous Wnt signaling in HEK293 cells has been amplified.

### Antibody-mediated Potentiation of Wnt Signaling Involves LRP6 Dimerization

**Figure 4:**
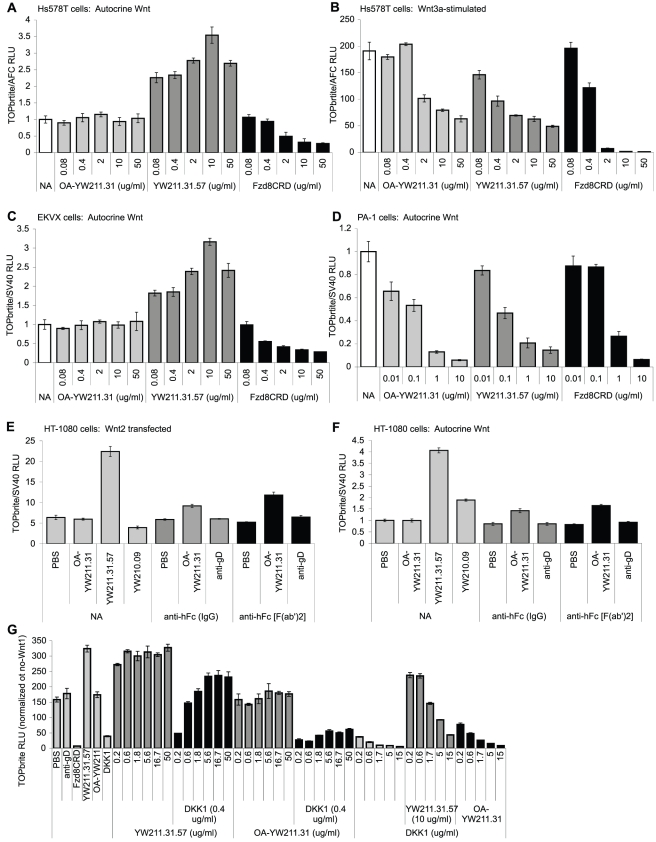
LRP6 antibody bivalency mediates potentiation of Wnt signaling. (A–D) One-armed (OA) YW211.31 antibody retains inhibitory activity similar in potency to the whole IgG against Wnt3a-induced signaling in Hs578T cells stably integrated with Wnt luciferase reporter (B) and against autocrine Wnt signaling in PA-1 cells transfected with reporter (D). The one-armed antibody lacks the potentiating effect of the intact YW211.31.57 antibody on autocrine Wnt signaling in Hs578T (A) or EKVX (C) cells. (E–F) Crosslinking of the one-armed YW211.31 antibody partially restores the potentiating activity observed with the whole antibody in HT-1080 cells for both autocrine Wnt signaling (F) and signaling induced by Wnt2 transfection (E). Similar results are observed with crosslinking by either the whole IgG or F(ab′)_2_ fragments of anti-human-Fc antibodies (NA indicates no crosslinking antibody added). All antibodies were used at 1 µg/ml. (G) HEK293 cells stably integrated with Wnt luciferase reporter were transfected for Wnt1 expression and treated with either 0.4 µg/ml DKK1 protein and increasing concentrations of YW211.31 intact or one-armed antibody, or with 10 µg/ml antibody and increasing concentrations of DKK1. Single-protein control treatments use 10 µg/ml antibody, 0.4 µg/ml DKK1, or 10 µg/ml Fzd8CRD-Fc. Wnt luciferase units were normalized to total protein concentration of the lysate, and additionally normalized to signal in cells not transfected for Wnt1 expression.

Potentiation of autocrine Wnt signaling by YW211.31 antibody requires avidity effects, presumably through LRP6 dimerization. A monovalent recombinant one-armed YW211.31.62 antibody (Figure 4A–4C) and the Fab fragment of YW211.31.62 (data not shown) exhibit no potentiation of autocrine Wnt signaling in EKVX and Hs578T cells at concentrations that inhibit Wnt3a-induced signaling in these cell lines. In contrast, the one-armed YW211.31 antibody and Fab fragment inhibit both autocrine Wnt and Wnt3a-induced signaling in the PA-1 cell line with similar potency to the intact IgG antibody (Figure 4D). As expected, the YW211.31.57 whole antibody, but not the one-armed antibody, is able to induce aggregation of differentially tagged LRP6 proteins at the surface of HEK293 cells (Figure S3).

To test whether crosslinking of the one-armed YW211.31 antibody would restore the Wnt potentiation function of the whole IgG molecule, we used the HT-1080 cell line that exhibits autocrine Wnt and Wnt2-induced signaling potentiated by YW211.31.57 antibody (Figure 4E and 4F). The one-armed YW211.31 antibody has no effect on autocrine Wnt signaling or signaling induced by Wnt2 transfection. HT-1080 cells also display Apomab antibody-induced apoptosis that is enhanced by Fc crosslinking (Figure S4) [26]. Under crosslinking conditions with anti-Fc antibodies that augment Apomab-mediated apoptosis, we find that crosslinking of the one-armed antibody partially reconstitutes the potentiation of both autocrine Wnt and Wnt2 signaling observed with the YW211.31.57 whole antibody (Figure 4E and 4F).

Inhibiting the activity of extracellular LRP6 antagonists such as DKK1 can also potentiate Wnt signaling [27]. Exogenous DKK1 protein partially inhibits Wnt1-induced signaling in HEK293 cells, and YW211.31.57 antibody can neutralize this antagonism and even weakly potentiate signaling at high enough concentration in the presence of DKK1 protein (Figure 4G). In contrast, one-armed YW211.31 antibody only very weakly and partially counteracts DKK1 antagonism of Wnt1 signaling at these same concentrations. YW211.31.57 whole antibody effectively antagonizes DKK1 activity at all DKK1 concentrations tested, whereas the one-armed antibody has minimal or no effect even at low DKK1 concentration. While we have not demonstrated that that endogenous DKK1 is present and active in HEK293 cells, the potent antagonism of exogenous DKK1 activity observed with the whole but not the one-armed YW211.31 antibody may contribute to the Wnt-potentiating activity specific to the whole antibody. Alternatively, since DKK1 protein was not able to inhibit completely the Wnt1-induced signaling in this assay, it is also possible that the intact YW211.31 antibody simply potentiates the remaining signal through LRP6 dimerization.

### Wnt Signaling Antagonism Predominates in LRP6 Antibody Combinations

**Figure 5:**
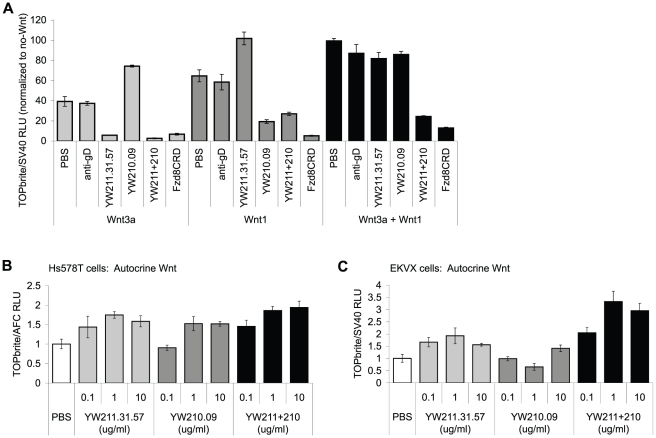
LRP6 antibody combinations can inhibit signaling induced by multiple Wnt isoforms. (A) The combination of YW211.31.57 and YW210.09 antibodies inhibits signaling in HEK293 cells stably integrated with Wnt luciferase reporter that have been transfected with expression constructs for either Wnt3a, Wnt1, or both Wnt3a and Wnt1. All antibodies and proteins were used at 10 µg/ml each. Treatment with either YW211.31.57 or YW210.09 antibody does not significantly change the level of signaling induced by the combination of both Wnt isoforms, possibly because signaling by one Wnt is antagonized while the other Wnt is potentiated. (B–C) As with individual antibody treatments, the combination of YW211.31.57 and YW210.09 antibodies potentiates autocrine Wnt signaling in Hs578T (B) or EKVX (C) cells.

Collectively, our results suggest that Wnt3a and Wnt3 bind within the E3-E4 region of LRP6 and are inhibited from binding by YW211.31.57 antibody. Wnt isoforms in the Wnt1 class are predicted to bind the E1-E2 region, and this binding may be blocked by YW210.09 antibody. In this model, potentiation of Wnt signaling would occur when both a Wnt isoform and an antibody are able to bind the same LRP6 molecule, presumably requiring recruitment of FZD by the Wnt and LRP6 dimerization by the antibody. This model predicts that the combination of both antibodies would inhibit signaling induced by either class of Wnt isoforms since, although LRP6 dimerization would likely still occur, Wnt binding would be blocked by one or the other antibody. As predicted, treating HEK293 cells simultaneously with YW211.31.57 and YW210.09 antibodies inhibits signaling initiated by expression of either Wnt3a or Wnt1 (Figure 5A). We extended this analysis to three other Wnt isoforms in the Wnt1 class and found that the combination of YW211.31.57 and YW210.09 antibodies inhibits Wnt signaling to a similar extent as YW210.09 alone (Figure 3E). When both Wnt3a and Wnt1 are expressed simultaneously, neither antibody antagonizes Wnt signaling, but the combination of both antibodies does inhibit signaling (Figure 5A). This result can be explained if each antibody inhibits binding of only one Wnt isoform, and both antibodies are able to bind LRP6 molecules simultaneously to block both Wnt binding sites.

Wnt isoforms in the third class (Wnt7a, 7b, and 10a) that are not antagonized by YW211.31.57 or YW210.09 antibody might bind to a site on LRP6 not blocked by either antibody or, alternatively, they may have the ability to bind to either of the Wnt-binding sites defined by the antibodies. For each of these Wnt isoforms, the combination of both antibodies also does not inhibit their signaling, but rather potentiates or does not affect their activity, suggesting that these Wnt isoforms can bind a site that is distinct from the YW211.31.57 and YW210.09 epitopes (Figure 3E).

The observed activities of the YW211.31.57 and YW210.09 antibody combination on Wnt signaling induced by exogenous Wnt isoforms also extend to endogenous, autocrine Wnt signaling. In PA-1 (Figure 1D) and Ntera-2 (data not shown) cell lines, in which YW211.31.57 antibody inhibits and YW210.09 potentiates autocrine Wnt signaling, the antibody combination inhibits signaling. In Hs578T and EKVX cells, where both antibodies potentiate autocrine Wnt signaling, the antibody combination also potentiates (Figure 5B and 5C).

### LRP6 Antibodies Differentially Inhibit Wnt Binding to Multiple Sites

**Figure 6:**
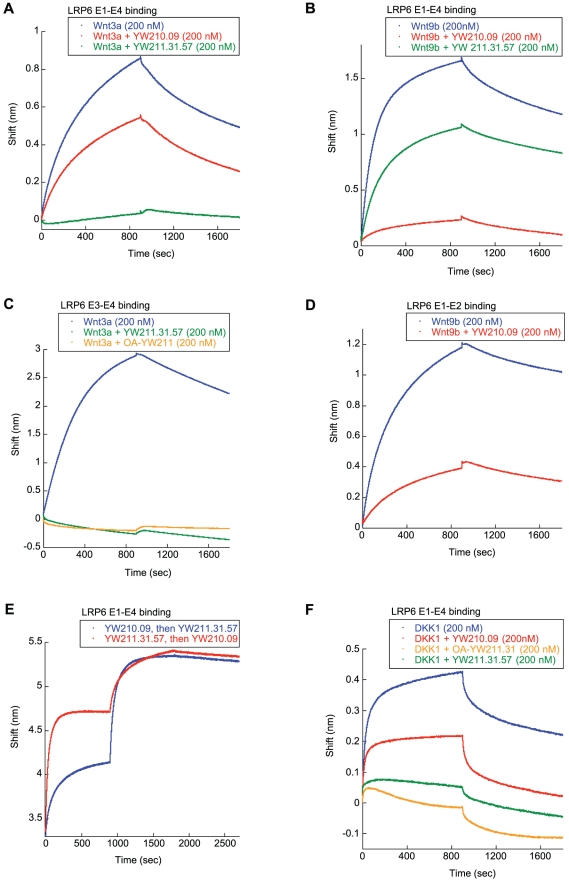
LRP6 antibodies block binding of Wnt isoforms to different sites. (A, B) Biolayer interferometry with biotinylated LRP6 E1-E4 protein immobilized on Streptavidin biosensors indicates that YW211.31.57 antibody inhibits binding of Wnt3a, but not Wnt9b, protein to LRP6 (A), and YW210.09 antibody inhibits only Wnt9b binding (B). Antibody binding to LRP6 protein was allowed to reach equilibrium, and the subsequent wavelength shift in the interference pattern is shown for association and dissociation phases of Wnt protein binding. (C, D) Smaller, non-overlapping fragments of LRP6 separate the binding of different antibodies and Wnt isoforms. Wnt3a binds to the E3-E4 region, and this interaction is blocked by either the intact or one-armed (OA) YW211.31 antibody (C). YW210.09 antibody binds the LRP6 E1-E2 protein fragment and competes with Wnt9b binding (D). (E) YW211.31.57 and YW210.09 antibodies can bind together to immobilized LRP6 E1-E4 protein when added sequentially in either order, confirming separate epitopes. (F) When either of these antibodies, or the one-armed YW211.31 antibody, is bound to LRP6 E1-E4 protein, DKK1 protein binding to LRP6 is antagonized.

The reciprocal activities of the two LRP6 antibodies suggest that YW211.31.57 and YW210.09 interact with distinct Wnt binding sites on LRP6, and that Wnt binding was competed by the antagonist antibody but allowed by the potentiating antibody. Bourhis *et al*. (2010) previously developed a biolayer interferometry assay that measures purified Wnt proteins binding to purified, immobilized LRP6 extracellular domain protein fragments [14]. This assay demonstrated that Wnt3a binds to the E3-E4 region of LRP6, where the epitope for YW211.31.57 antibody resides, and Wnt9b (in the same class as Wnt1 for antibody interactions) binds only to the E1-E2 region, where YW210.09 antibody also binds. Here we show that YW211.31.57 antibody, but not YW210.09, inhibits binding of Wnt3a to the LRP6 E1-E4 protein fragment (Figure 6A). Conversely, YW210.09 but not YW211.31.57 inhibits Wnt9b binding to E1-E4 (Figure 6B). Antibody-mediated inhibition of Wnt binding can also be detected using the smaller Wnt-binding fragments, E3-E4 for Wnt3a and E1-E2 for Wnt9b (Figure 6C and 6D). The one-armed YW211.31 antibody can also inhibit Wnt3a binding to the E3-E4 fragment (Figure 6C). Also, YW211.31.57 and YW210.09 can be bound sequentially to LRP6 E1-E4 protein without competition and when added in either order (Figure 6E). Competition for binding between YW211.31.57 antibody and only Wnt3a, and between YW210.09 and only Wnt9b, at different sites on LRP6 protein correlate with the inhibitory activity of each antibody against signaling by a specific Wnt isoform.

Bourhis *et al*. (2010) showed by biolayer interferometry that purified DKK1 protein could bind both E3-E4 and E1-E2 fragments of LRP6, and that this interaction could inhibit binding of Wnt3a and Wnt9b to these respective protein regions [14]. We find that YW211.31.57 and YW210.09 antibodies can each inhibit DKK1 binding to LRP6 E1-E4 protein (Figure 6F). As expected, YW211.31.57 antibody also inhibits DKK1 binding to LRP6 E3-E4 protein, and YW210.09 antibody blocks binding of DKK1 to the E1-E2 fragment (data not shown). The one-armed YW211.31 antibody fully retains this inhibitory activity, even though it cannot potentiate Wnt signaling nor significantly antagonize exogenous DKK1 activity on Wnt signaling in cells. This result suggests that the ability to block DKK1 binding to LRP6, at least *in vitro*, does not contribute significantly to antibody-mediated potentiation of Wnt signaling, or to antagonism of DKK1 activity. Rather, dimerization of coreceptor that is also able to bind Wnt primarily mediates both these activities.

### LRP6 Antibodies are Active on Wnt-driven Tumors and Bone Formation

**Figure 7:**
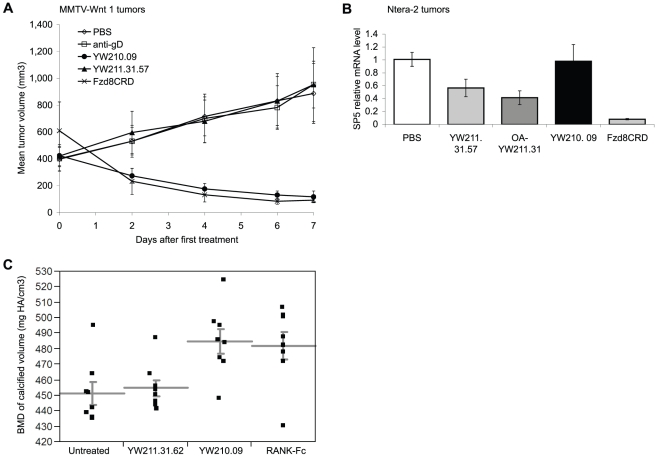
Wnt-driven signaling and growth in tumors, and mineral density in bone, are modulated by specific LRP6 antibodies. (A) MMTV-Wnt1 allograft tumors show regression of growth when mice are treated with YW210.09 antibody, similar to that observed with Fzd8CRD-Fc protein. YW211.31.57 antibody did not alter tumor growth under these conditions compared to control buffer (PBS) or anti-gD antibody treatment. Mice were administered 30 mg/kg of antibody or protein every two days (arrowheads). (B) Ntera-2 xenograft tumors display reduced expression of SP5 mRNA by qPCR analysis in mice treated with intact or one-armed (OA) YW211.31 antibody, but not with YW210.09 antibody. SP5 mRNA levels were normalized to GAPDH mRNA levels within the same tumor, and additionally normalized to PBS-treated tumors. All treatments except YW210.09 display p-value <0.005 by ANOVA compared to PBS control. (C) YW210.09, but not YW211.31.62, antibody treatment of mouse calvaria explants in culture significantly increases bone mineral density (BMD) of calcified parietal bone, similar to treatment with RANK-Fc protein. All treatments were 10 µg/ml antibody or protein for 7 d. Data points represent eight calvaria halves from four mice for each treatment group; mean and standard error of the mean are shown as horizontal and vertical lines, respectively. Only YW210.09 and RANK-Fc treatments differ significantly from untreated samples with p-values less than 0.01 and 0.05, respectively, by Dunnett's test (<0.05 for both by t-test).

To begin to explore the anti-tumor therapeutic efficacy of the LRP6 antibodies, we treated two models of Wnt ligand-driven tumors, MMTV-Wnt1 transgenic mammary tumor allografts dependent on Wnt1 expression and Ntera-2 human teratocarinoma xenografts driven by autocrine Wnt signaling of an unknown Wnt isoform [21]. For MMTV-Wnt1 tumors, rapid and sustained tumor regression was observed with YW210.09 antibody treatment, similar to Fzd8CRD-Fc protein, whereas no effect on tumor growth was evident with YW211.31.57 antibody (Figure 7A). These results are consistent with the antibody effects described above on Wnt target gene expression for MMTV-Wnt1 tumor cells treated in tissue culture.

For Ntera-2 xenograft tumors, RNA extracted from tumors treated with antibodies YW211.31.57, one-armed YW211.31, or the combination of YW211.31.57 and YW210.09 reveals reduced expression of Wnt target gene SP5 to 41–57% the level of PBS-treated tumors, whereas Fzd8CRD-Fc protein treatment reduced SP5 expression to 8.0% (Figure 7B). Axin2 expression was reduced to only 56% by Fzd8CRD-Fc, and no significant changes in Axin2 expression were detected with any of the antibody treatments (data not shown). YW210.09 antibody treatment did not significantly affect expression of either SP5 or Axin2. Serum samples assayed for inhibition or potentiation of Wnt3a-induced signaling in HEK293 cells confirm that injected antibodies and protein retained at least some activity *in vivo* throughout the 16-h exposure (data not shown).

Since activation or potentiation of Wnt signaling can increase bone mass by enhancing osteoblast differentiation and function, as well as by inhibiting osteoclast differentiation indirectly [28], we tested the activity of LRP6 antibodies on mouse calvarial bones in organotypic culture. Microdissected calvaria explants were cultured with antibody or RANK-Fc protein, and then parietal bone volume and density were analyzed by micro-computed tomography. Using histogram analysis of control samples, X-ray attenuation ranges were defined for calcified (bone) and non-calcified (cartilage) tissues. Treatment with YW210.09 antibody significantly increased the mean bone mineral density (BMD) of calcified parietal bone by 7.4%, similar to the 6.8% increase observed with RANK-Fc treatment to inhibit osteoclast differentiation (Figure 7C) [29]. Treatment with YW211.31.62 antibody did not significantly change calcified parietal BMD. The volume of total parietal bone region (calcified and non-calcified) and the proportion of calcified bone in this region were not significantly changed by antibody or RANK-Fc treatments, suggesting that YW210.09 antibody may enhance mineralization without gross changes in cell proliferation (data not shown).

## Discussion

**Figure 8:**
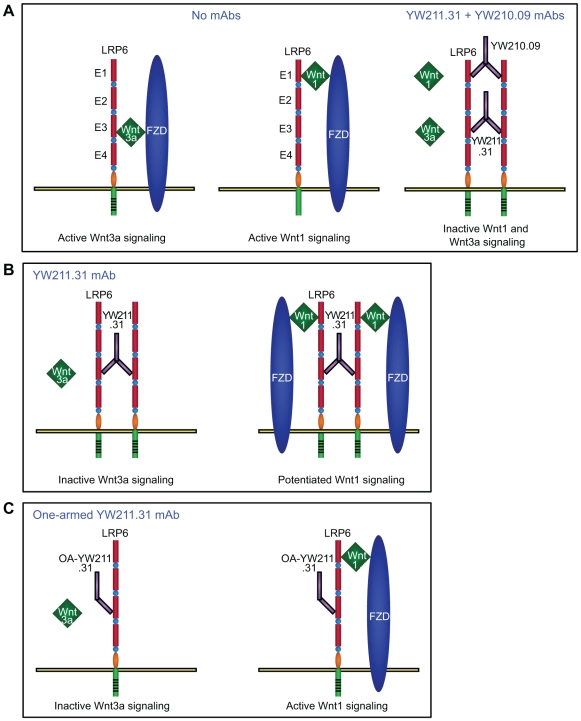
Model of LRP6 interactions with antibodies and Wnt isoforms. (A–B) Wnt3a and Wnt3 induce signaling by binding the E3-E4 region of LRP6 and recruiting FZD into the complex, whereas Wnt1, Wnt9b, and other Wnt isoforms in this class (Figure 3C) bind the E1-E2 region. YW211.31.57 and YW210.09 antibodies recognize LRP6 regions E3-E4 and E1-E2, respectively, to inhibit signaling by only the Wnt isoforms that bind these regions. Both antibodies can bind an LRP6 molecule simultaneously to antagonize signaling in the presence of Wnt isoforms from both classes. Each antibody can potentiate signaling by the class of Wnt isoforms that bind regions outside its epitope, through crosslinking coreceptors and allowing Wnt to recruit FZD. (C) A one-armed antibody can inhibit signaling by blocking Wnt binding, but cannot potentiate since it does not dimerize LPR6.

We have identified antibodies against LRP6 that can exert both antagonist and potentiating activities on β-catenin signaling, and demonstrate that these activities depend on different interactions between Wnt isoforms and the coreceptor (Figure 8). Since all antibodies screened that antagonize signaling in Wnt3a-stimulated HEK293 cells also inhibit Wnt3a stimulation in all other cell lines tested, and also inhibit autocrine Wnt signaling in teratocarcinoma cell lines, it was intriguing that these antibodies potentiate autocrine Wnt signaling in the other nine cell lines tested. In addition, the YW210.09 antibody potentiates Wnt3a signaling in all cell lines tested and enhances autocrine Wnt signaling in 7 cell lines, but it inhibits endogenous signaling in 3 other lines. We discovered that introduction of different Wnt isoforms into the same cell line determines the activity of the LRP6 antibodies, and that Wnt3a antagonist and potentiating antibodies also have reciprocal effects on most other Wnt proteins. Based on their functional interaction with two LRP6 antibodies, the 14 Wnt isoforms tested can be grouped into three classes: Wnt3 and Wnt3a are inhibited by YW211.31 and potentiated by YW210.09; Wnts 1, 2, 2b, 6, 8a, 9a, 9b, and 10b are potentiated by YW211.31 and antagonized by YW210.09; and Wnts 4, 7a, 7b, and 10a are potentiated by YW211.31 and not inhibited by YW210.09 (Figure 3C). These classifications do not obviously correspond to the proposed phylogeny of Wnt genes, although the Wnt3/3a subfamily is the most evolutionarily divergent [30].

Chimeric proteins that constitutively activate signaling by fusing different Wnt and FZD isoforms confirm that the activities of the antibodies are determined by the isoform of Wnt, and not FZD. Chimeric protein fusions of Wnt isoforms with LRP6, but not FZD, are insensitive to inhibition by the LRP6 antibodies, suggesting that antagonism may be mediated by blocking ligand-coreceptor interactions. This is confirmed by binding studies *in vitro* for Wnt3a and YW211.31 antibody, which bind competitively within the E3-E4 region of LRP6, and for Wnt9b and YW210.09 antibody, which compete for binding within the E1-E2 region. The epitopes of the two LRP6 antibodies each define a binding site for a different class of Wnt isoforms, one within the E1-E2 and one within the E3-E4 domain. Our data suggest that there may be at least a third binding site for Wnt isoforms that are not inhibited by either antibody or their combination, and it seems likely each of the four repeat domains binds a different subset of Wnt isoforms. This modular organization might allow for structural divergence of different Wnts and their binding sites to accommodate differential regulation by Wnt-binding and coreceptor-binding antagonists such as SFRP and DKK protein isoforms, respectively.

Antibody-mediated Wnt potentiation requires coreceptor dimerization, since one-armed and Fab antibody formats fail to enhance Wnt signaling unless crosslinked. In addition, our cell-based and biochemical data indicate that Wnt binding to crosslinked LRP6 is also necessary for potentiation of signaling, presumably reflecting a requirement for Wnt-mediated recruitment of FZD into the complex. A small fraction of overexpressed LRP6 can be identified as a homodimer at the cell surface, and dimerization requires the extracellular domain, however it is not clear whether this contributes to either Wnt-dependent or -independent β-catenin signaling induced by LRP6 overexpression [18]. Deletion of the LRP6 extracellular domain potently activates signaling in a Wnt-independent manner, and forced extracellular dimerization of this recombinant protein by different methods can either enhance or inhibit this activity [18], [19]. Wnt induces LRP6 aggregation and phosphorylation at the plasma membrane that both require the homo-oligomerization function of intracellular DVL protein [7]. These large aggregates also contain Axin and GSK3, and likely inhibit β-catenin degradation. It will be interesting to determine whether LRP6 dimerization by antibodies or other extracellular agents facilitates the aggregation function of DVL, and whether coreceptor dimerization normally plays a functional role in Wnt signaling.

LRP6 antibodies might also potentiate Wnt signaling by inhibiting binding of antagonists such as DKK isoforms and SOST. We show that YW211.31 antibody can antagonize the activity of exogenous DKK1 protein, and that the one-armed YW211.31 antibody, which does not potentiate Wnt, retains only very weak DKK1 antagonism. However, both the whole IgG and the one-armed antibody show potent and similar activity in blocking DKK1 binding to LRP6 protein *in vitro*. Since antibody inhibition of DKK1 interaction with LRP6 does not necessarily confer Wnt potentiating activity, and DKK1 antagonism seems to require LRP6 dimerization, the inhibition of DKK1 activity is likely mediated predominantly by potentiation of residual signaling from Wnt bound to coreceptor. Two other groups have reported identification of LRP6 antibodies that bind the E1-E2 domains and potentiate Wnt3a signaling, although they did not test effects on signaling induced by other Wnt isoforms [31], [32]. Analogous to YW211.31 antibody, one of these antibodies inhibits exogenous DKK1 antagonism of Wnt3a signaling, and also blocks DKK1 binding to LRP6 *in vitro* [31]. A Fab fragment of this antibody fails to potentiate Wnt3a signaling and antagonizes exogenous DKK1 activity much less potently. Apparently, inhibition of DKK1 binding to LPR6 within the E1 domain, recognized by this antibody, or the E3-E4 domain, bound by YW211.31 antibody, contributes only weakly to the antagonism of DKK1 activity by these antibodies.

In Wnt-driven xenograft and allograft tumor models, the LRP6 antibodies inhibit Wnt signaling and tumor growth in a Wnt isoform-specific manner predicted by their properties characterized in cell culture. It is not clear why we failed to observe enhancement of signaling or growth in tumors by the antibodies, and it is possible in the MMTV-Wnt1 model that Wnt signaling levels are already maximal. We did detect a significant increase in bone mineral density in the calvaria explant model with YW210.09 antibody, and it will be important to confirm that this is mediated by potentiation of Wnt signaling in osteoblasts. While β-catenin dependent Wnt signaling is known to be required for intramembranous ossification of skull bones during embryogenesis, the specific Wnt isoforms involved have not been identified, although exogenous Wnt3a treatment can increase cranial osteoblast differentiation and enhance bone mineralization [33], [34]. Interestingly, the activities of the LRP6 antibodies on endogenous Wnt signaling and biological processes can be used to predict a subset of Wnt isoforms active in specific models and systems. RNAi of candidate Wnts identifies Wnt1, Wnt2, Wnt3, and Wnt7b as the specific isoforms mediating autocrine Wnt signaling in tumor cell lines M14 (melanoma), NCI-H23 (NSCLC), PA-1 (teratocarcinoma), and Hs578T (breast), respectively. While mutations in Wnt genes have been linked to several human diseases (http://www.stanford.edu/~rnusse/diseases/Humangeneticdis.htm), further identification of specific Wnt isoforms mediating tumorigenesis in different cancer types, skeletal diseases, and stem cell maintenance will clearly be critical for realizing the therapeutic potential of Wnt coreceptor antibodies. With this knowledge, LRP5/6 antibodies and their alternative formats, including one-armed and bipecific antibodies, may enable finely tuned antagonism and potentiation of the many tissue-specific roles that the 19 human Wnt isoforms have evolved to perform.

## Materials and Methods

### Ethics Statement

All experiments using mice were approve by the Genentech Institutional Animal Care and Use Committee (protocol numbers 07-0194G, 09-1648, and TH10-0841).

### Cell Culture and Cell Assays

Cell lines EKVX and M14 were obtained from National Cancer Institute (National Institutes of Health, Frederick, MD) and grown in RPMI-1640 medium supplemented with 10% fetal bovine serum and 2 mM glutamine. JHH-1 cells were obtained from Japan Health Science Foundation (Health Science Research Resources Bank, Osaka, Japan) and grown in Williams' Medium E with the same supplements. All other cell lines were purchased from American Type Culture Collection (ATCC) and maintained as recommended.

Luciferase reporter assays were performed as previously described [21] using TOPglow (Upstate) or TOPbrite [36] firefly luciferase Wnt reporter, or pRL-SV40 Renilla luciferase (Promega) plasmid. Cells were treated with antibodies for 16–20 h, starting 24 h after transfection. Either purified Wnt3a protein was added to cells starting 1 h after initiating antibody treatment, or Wnt expression constructs were co-transfected with luciferase reporters. Firefly luciferase levels were normalized for transfection efficiency to Renilla luciferase levels, and the relative luciferase units (RLU) were additionally normalized to the level in cells not stimulated with Wnt.

HEK293 and Hs578T cell lines stably integrated with TOPbrite reporter were selected for hygromycin resistance. Expression of Wnt luciferase reporter was normalized to cell number based on stably integrated SV40-driven Renilla luciferase for HEK293 cells or on the MultiTox-Fluor cell viability assay (AFC fluorescence; Promega) for Hs578T cells.

For quantitative real-time PCR (qPCR) expression analysis, RNA was isolated from cells using the RNeasy kit (QIAGEN), and reactions were performed with the TaqMan One-Step RT-PCR Master Mix Reagents Kit (Applied Biosystems) on the 7900 HT Fast Real-Time PCR System (Applied Biosystems). Relative RNA levels were calculated using the ΔΔCt method and normalized to human GAPDH or mouse Rpl19 RNA levels within the same sample, and additionally normalized to samples from cells with no addition (NA) of Wnt3a, antibody, or other proteins. The primer and probe sets, listed 5′ to 3′ for forward primer, reverse primer, and probe sequences, respectively, are SP5: AATGCTGCTGAACTGAATAGAAA, AACCGGTCCTAGCGAAAA, CCGAGCACTGTTTCAAATCTCCCA; ZNRF3: TGAGAGTGTGACATTGTTGGAA, GTAAAATCTGTGTGCAATTATCATGT, AATCATTGAAAATGACTAACACAAGACCCTGTAAAT; mouse Mmp7: TGAGGACGCAGGAGTGAA, CCCAGAGAGTGGCCAAAT, CCTGTTTGCTGCCACCCATGA. Primers and probes used for human APCDD1, AXIN2, GAD1, LEFTY2, and SAX1, and for mouse Rpl19 and Axin2, were previously described [21], [37]. GAPDH primers and probe were purchased from Applied Biosystems. For reporter gene and qPCR assays, all figures represent the mean and standard deviation of three or four experimental replicates.

For Western analysis, 1.2×10^6^ HEK293 cells were seeded onto 10-cm dishes and treated 3 days later with 10 µg/ml antibody, 2.5 µg/ml DKK1 (R&D Systems), or 2.5 µg/ml Fzd8CRD-Fc protein [21] for 1 h before adding 0.2 µg/ml Wnt3a protein for an additional 1 h. Cells were washed twice with cold PBS and lysed in 0.5 ml SJC lysis buffer [36] on ice. For sequential affinity purification of LRP6 prior to Western analysis, HEK293 cells were transfected with 0.33 µg/ml each of LRP6-flag and LRP6-HA expression constructs using FuGENE 6 reagent (Roche), treated 1 day later with 1 µg/ml antibody for 1 h on ice, washed with PBS, and lysed in SJC buffer containing 1% *n*-dodecyl-β-D-maltoside (DDM). Cold cell lysate was homogenized by five passes through a 28-gauge needle and clarified by centrifugation at 25,000 x *g* for 15 min. EZview Red ANTI-FLAG M2 affinity gel (60 µl settled resin; Sigma-Aldrich) was added to 500 µg of lysate protein in 500 µl lysis buffer and rotated overnight at 4°C. Affinity gel was washed twice in lysis buffer, twice in TBS (10 mM Tris HCl, 150 mM NaCl, pH 7.4) containing 1% DDM, and subjected to two sequential affinity elutions, each using 300 µl of TBS containing 1% DDM and 200 µg/ml FLAG peptide (Sigma-Aldrich) for 30 min at 4°C. Pooled eluate was combined and incubated with nProtein A Sepharose and Protein G Sepharose Fast Flow (25 µl of a 1∶1 mix of settled resins; GE Healthcare) overnight at 4°C. Sepharose was washed three times with TBS containing 1% DDM, once with TBS, and incubated at 90°C for 10 min in 60 µl NuPAGE LDS sample buffer and sample reducing agent (Invitrogen). 20 µg of protein, or 20 µl of affinity purified sample, was electrophoretically resolved on a denaturing SDS-polyacrylamide gel (4–12%), transferred to nitrocellulose membrane, and probed with antibodies against phospho- and total LRP6 (Cell Signaling Technology), β-catenin (BD Transduction Laboratories), β-actin (Sigma-Aldrich), GAPDH (Millipore), HA (Covance), and FLAG (Sigma-Aldrich). Proteins were visualized using infrared labeled secondary antibodies (Rockland Immunochemicals) and Odyssey imager (LI-COR).

For RNAi experiments, ON-TARGETplus double-stranded siRNA oligomers (Thermo Scientific) were resuspended and used for 72-hour transfection according to the manufacturer's instructions with DharmaFECT 2 reagent at a final dilution of 1∶500, individual siRNAs at 20 nM, and pools of four siRNAs at 80 nM. Individual control siRNAs used for LRP5, LRP6, and non-targeting were Thermo Scientific catalog numbers J-003844-06, J-003845-11, and D-001810-01-05, respectively.

For fluorescence-activated cell sorting (FACS), HEK293 cells stably integrated for LRP6 expression were harvested in PBS, washed with PBS containing 5 mM EDTA and 1% fetal bovine serum, and incubated for 1 h on ice with 0.5 µg/ml primary antibody, with or without pre-incubation with purified LRP6 E1-E4-His protein for 1 h, in 96-well format at 500,000 cells per well. Cells were washed twice with the same buffer, incubated for 1 h on ice with 2.5 µg/ml R-phycoerythrin-conjugated anti-human IgG (Jackson ImmunoResearch Laboratories), washed three times, and suspended in buffer containing 0.5 µg/ml propidium iodide. Analysis was performed using a FACS Calibur instrument (Becton Dickinson) and FlowJo software (Tree Star), using appropriate gates to exclude cellular debris and aggregates.

Wnt chimera constructs were made by cloning full-length Wnt1 or Wnt3a upstream of full-length FZD4, FZD5, or LRP6 in pRK5 expression vector. The 24-amino acid linker (GGGSGGGT)_3_ was inserted between Wnt and FZD or LRP6 sequences [19].

### LRP6 Antibody Generation and Testing

Human LRP6 cDNA fragments encoding regions E1-E2 (amino acids A16-R657) and E3-E4 (amino acids A624-G1254) were separately cloned into a mammalian expression vector containing the HSV signal sequence and human IgG Fc region as a protein tag. LRP6 E1-E2-Fc and E3-E4-Fc proteins were expressed in CHO cells by transient transfection, purified by Protein A affinity chromatography, and used individually to screen human synthetic antibody phage libraries. After selection on immobilized LRP6 protein, phage clones were isolated and confirmed by phage ELISA for binding to the LRP6-Fc fusion protein fragment and not anther Fc fusion protein. Phage Fab clones were then reformatted for expression as human IgG1 monoclonal antibodies. Twenty-four unique clones against LRP6 E1-E2-Fc and 22 clones against E3-E4-Fc were transfected and transiently expressed in HEK293 cells, and IgG protein was purified by affinity chromatography. Subsequent large-scale antibody preparations were produced by transient transfection in CHO cells.

For affinity maturation of YW211.31 antibody, three different combinations of CDR loops (H1/L3, H2/L3, and H3/L3) were targeted for randomization in separate libraries by soft-randomizing selected residues. In addition, the L1/L2/L3 CDR combination was targeted for hard randomization. In the first round of selection, phage from the randomized libraries were selected with immobilized LRP6 E3-E4-His protein, followed by five rounds of solution-phase sorting in which the concentration of E3-E4-His was gradually reduced from 300 nM to 0.5 nM, and a 100-fold excess of E3-4-Fc protein was added to deplete antibodies with faster dissociation rates. Eleven phage clones were purified, and all showed improved affinity for LRP3 E3-E4 as determined by phage competition ELISA. The sequence of these clones displayed 1 to 6 amino acid changes in CDR-H1, CDR-H3, and CDR-L3.

The one-armed YW211.31 antibody variant was produced in *E. coli* by co-expressing the YW211.31.62 heavy and light chains with a truncated Fc domain using ‘knobs-into-holes’ engineering technology [38]. For antibody cross-linking, Fc-specific goat-anti-human IgG antibody or F(ab′)2 fragment (Sigma-Aldrich) was incubated with one-armed YW211.31 or Apomab antibody for 1 h before adding the mixture to cells.

For binding affinity determination of antibodies, SPR measurement with a BIAcore™-3000 instrument was used. To measure affinity between LRP6 antibodies and LRP6 E1-E2-His protein (expressed in insect cells) or LRP6 E3-E4-His protein (expressed in CHO cells), anti-LRP6 human IgG was captured by CM5 biosensor chips coated mouse anti-human IgG to achieve approximately 100 response units. For kinetics measurements, two-fold serial dilutions of LRP6 E1-E2-His (250 nM to 3.9 nM) or E3-E4-His (31.2 nM to 0.49 nM) protein was injected in PBS with 0.05% Tween 20 at 25°C with a flow rate of 30 µl/min. Association rates (*k*_on_) and dissociation rates (*k*_off_) were calculated using a simple one-to-one Langmuir binding model (BIAcore Evaluation Software version 3.2). The equilibrium dissociation constant (*K*_D_) was calculated as the ratio *k*_off_/*k*_on_.

### LRP6 Protein Binding Assay

Biolayer interferometry was performed as previously described [14]. Briefly, biotinylated, His-tagged LRP6 proteins were purified from baculovirus-infected insect cells using the AviTag system (GeneCopoeia). Binding kinetics were measured on the Octet RED System (ForteBio) using Streptavidin High Binding FA Biosensors loaded with 20 µg/ml LRP6 protein. Carrier-free purified human Wnt3a and mouse Wnt9b were obtained from R&D Systems, and purified DKK1 protein was produced as previously described [14].

### Tumor and Bone Studies

Tumors from MMTV-Wnt1 transgenic mice were passaged in mammary fat pads of C57BL/6 mice, mechanically and enzymatically dissociated, resuspended in Matrigel and Hank's Balanced Salt Solution (HBSS), and injected into the mammary fat pad of athymic NCr nude mice (Taconic). Treatments were initiated once tumor volumes reached 250–800 mm^3^. For each treatment group, ten mice were administered 30 mg/kg of antibody or protein intraperitoneally (IP) every two days. Tumor volume was analyzed using caliper measurement.

Ntera-2 xenograft tumor growth and in vivo studies were performed as previously described [21]. Briefly, NU/NU athymic nude mice (Charles River) were injected subcutaneously with 10 million Ntera-2 cells (in 50% Matrigel in HBSS) per mouse, divided into groups of four or five animals once mean tumor volumes reached 535–595 mm^3^, and injected with a single IP dose of 100 mg/kg antibody or 30 mg/kg Fzd8CRD-Fc protein. Tumor and blood serum samples were collected 16 h after treatment. Tumors were homogenized using the TissueLyser system (QIAGEN), and RNA was extracted using the RNeasy kit (QIAGEN).

Calvariae were harvested and cultured as described previously [39]. Briefly, calvariae were dissected from 2-day-old mouse pups, cut into halves, and separated from dura mater, vessels, and scalp. Calvariae were cultured in tissue culture plates in BGJb medium supplemented with 0.1% bovine serum albumin and 100 U/ml each of penicillin and streptomycin for 1 day before treating with 10 µg/ml antibody or protein for 7 days. The bones were cultured in a humidified atmosphere of 5% CO_2_ at 37°C. Mouse calvariae were imaged with a µCT 40 (SCANCO Medical, Basserdorf, Switzerland) X-ray micro-computer tomography (micro-CT) system with the following parameters: X-ray tube energy level of 45 kV, current of 177 µA, integration time of 300 msec, and 2000 projections. Axial images were obtained at an isotropic resolution of 6 µm. A hydroxyapatite (HA) phantom was used for calibration. Micro-CT scans were analyzed with Analyze (AnalyzeDirect Inc., Lenexa, KS, USA). Maximum-intensity projections and three-dimensional surface renderings in the transverse plane were created for each sample. Parietal bone borders were manually defined within micro-CT images in order to segment the parietal region. Within this region, sample volume and mean bone mineral density (BMD) were calculated. A threshold of 0.3 gm-HA/cm^3^ was applied to the region in order to calculate the mean BMD of only calcified tissue within the region. The threshold was also used to calculate percentage calcified volume of the parietal region by dividing the number of calcified voxels over the total voxels for the parietal region. The following parameters were analyzed for each sample: parietal region volume, BMD of calcified voxels of the parietal region, and parietal region percentage calcified.

## Supporting Information

Figure S1 LRP6 antibodies do not bind additional cell surface proteins. To block LRP6-dependent binding to live HEK293 cells stably expressing LRP6, the indicated antibodies were incubated with different concentrations of purified LRP6 E1-E4 protein and then used for indirect immunofluorescence flow cytometry. The relative fluorescence obtained with each antibody in the absence of competing soluble LRP6 protein was normalized to 100%. Greater than 50% reduction in cell binding is achieved at a 0.62-fold molar ratio of competing protein to antibody, and over 97% of binding is blocked by 5.6-fold molar excess of soluble LRP6 protein.
(0.34 MB EPS)

Figure S2 RNAi of candidate autocrine Wnts identifies isoforms mediating endogenous signaling in tumor cell lines. (A–D) Individual and pools of four siRNAs against Wnt isoforms were transfected into PA-1 (A), M14 (B), Hs578T (C), and NCI-H23 (D) cell lines stably integrated with Wnt luciferase reporter. Expression of luciferase is normalized to cell number and additionally normalized to levels in cells not transfected with siRNA. β-catenin, LRP6, and LRP5 RNAi, as well as Fzd8CRD-Fc protein (10 μg/ml) treatment, serve as controls for detection of autocrine Wnt signaling.
(0.45 MB EPS)

Figure S3 Whole but not one-armed antibody crosslinks LRP6 at the cell membrane. HEK293 cells transfected for expression of both flag- and HA-tagged LRP6 were treated with the indicated antibodies, and LRP6-flag was immunoprecipitated from cell lysates. After elution, LRP6-flag complexes bound to LRP6 antibodies were affinity purified using Protein A/G resin and subjected to Western analysis for crosslinked LRP6-HA. As previously reported [40], the higher-molecular-weight LRP6 species is predominant at the cell surface and accessible to antibodies. Control lanes represent transfection of empty expression vector, cell treatment with anti-gD antibody or with no antibody (NA), and whole-cell lysates (WCL) for LRP6-flag and -HA transfection or for HEK293 cells stably expressing LRP6-flag.
(1.23 MB EPS)

Figure S4 Apomab antibody crosslinking induces apoptosis in HT-1080 cells. We utilized the crosslinking-dependent proapoptosis activity of Apomab antibody to optimize experimental conditions for crosslinking receptors on HT-1080 cells by either the whole IgG or F(ab′)2 fragment of anti-human-Fc antibodies (NA indicates no crosslinking antibody added). Results are shown for 1 μg/ml of all antibodies. Cell viability was monitored after 17 h of antibody treatment using the MultiTox-Fluor assay (Promega).
(0.32 MB EPS)

## References

[1] Grigoryan T, Wend P, Klaus A, Birchmeier W (2008). Deciphering the function of canonical Wnt signals in development and disease: conditional loss- and gain-of-function mutations of beta-catenin in mice.. Genes Dev.

[2] van Amerongen R, Nusse R (2009). Towards an integrated view of Wnt signaling in development.. Development.

[3] Tamai K, Semenov M, Kato Y, Spokony R, Liu C (2000). LDL-receptor-related proteins in Wnt signal transduction.. Nature.

[4] Semënov MV, Tamai K, Brott BK, Kühl M, Sokol S (2001). Head inducer Dickkopf-1 is a ligand for Wnt coreceptor LRP6.. Curr Biol.

[5] Tamai K, Zeng X, Liu C, Zhang X, Harada Y (2004). A mechanism for Wnt coreceptor activation.. Mol Cell.

[6] Mi K, Dolan PJ, Johnson GV (2006). The low density lipoprotein receptor-related protein 6 interacts with glycogen synthase kinase 3 and attenuates activity.. J Biol Chem.

[7] Bilic J, Huang YL, Davidson G, Zimmermann T, Cruciat CM (2007). Wnt induces LRP6 signalosomes and promotes dishevelled-dependent LRP6 phosphorylation.. Science.

[8] Schwarz-Romond T, Metcalfe C, Bienz M (2007). Dynamic recruitment of axin by Dishevelled protein assemblies.. J Cell Sci.

[9] Cselenyi CS, Jernigan KK, Tahinci E, Thorne CA, Lee LA (2008). LRP6 transduces a canonical Wnt signal independently of Axin degradation by inhibiting GSK3's phosphorylation of beta-catenin.. Proc Natl Acad Sci U S A.

[10] Piao S, Lee SH, Kim H, Yum S, Stamos JL (2008). Direct inhibition of GSK3beta by the phosphorylated cytoplasmic domain of LRP6 in Wnt/beta-catenin signaling.. PLoS One.

[11] Zeng X, Huang H, Tamai K, Zhang X, Harada Y (2008). Initiation of Wnt signaling: control of Wnt coreceptor Lrp6 phosphorylation/activation via frizzled, dishevelled and axin functions.. Development.

[12] Wu G, Huang H, Garcia Abreu J, He X (2009). Inhibition of GSK3 phosphorylation of beta-catenin via phosphorylated PPPSPXS motifs of Wnt coreceptor LRP6.. PLoS One.

[13] Jeon H, Meng W, Takagi J, Eck MJ, Springer TA (2001). Implications for familial hypercholesterolemia from the structure of the LDL receptor YWTD-EGF domain pair.. Nat Struct Biol.

[14] Bourhis E, Tam C, Franke Y, Bazan JF, Ernst J (2010). Reconstitution of a Frizzled8-Wnt3a-LRP6 Signaling Complex Reveals Multiple Wnt and Dkk1 Binding Sites on LRP6.. J Biol Chem.

[15] Rebay I, Fleming RJ, Fehon RG, Cherbas L, Cherbas P (1991). Specific EGF repeats of Notch mediate interactions with Delta and Serrate: implications for Notch as a multifunctional receptor.. Cell.

[16] Davis-Smyth T, Chen H, Park J, Presta LG, Ferrara N (1996). The second immunoglobulin-like domain of the VEGF tyrosine kinase receptor Flt-1 determines ligand binding and may initiate a signal transduction cascade.. EMBO J.

[17] Cunningham SA, Stephan CC, Arrate MP, Ayer KG, Brock TA (1997). Identification of the extracellular domains of Flt-1 that mediate ligand interactions.. Biochem Biophys Res Commun.

[18] Liu G, Bafico A, Harris VK, Aaronson SA (2003). A novel mechanism for Wnt activation of canonical signaling through the LRP6 receptor.. Mol Cell Biol.

[19] Cong F, Schweizer L, Varmus H (2004). Wnt signals across the plasma membrane to activate the beta-catenin pathway by forming oligomers containing its receptors, Frizzled and LRP.. Development.

[20] Lee CV, Liang WC, Dennis MS, Eigenbrot C, Sidhu SS (2004). High-affinity human antibodies from phage-displayed synthetic Fab libraries with a single framework scaffold.. J Mol Biol.

[21] DeAlmeida VI, Miao L, Ernst JA, Koeppen H, Polakis P (2007). The soluble wnt receptor Frizzled8CRD-hFc inhibits the growth of teratocarcinomas in vivo.. Cancer Res.

[22] Bafico A, Liu G, Goldin L, Harris V, Aaronson SA (2004). An autocrine mechanism for constitutive Wnt pathway activation in human cancer cells.. Cancer Cell.

[23] Akiri G, Cherian MM, Vijayakumar S, Liu G, Bafico A (2009). Wnt pathway aberrations including autocrine Wnt activation occur at high frequency in human non-small-cell lung carcinoma.. Oncogene.

[24] Guo Y, Xie J, Rubin E, Tang YX, Lin F (2008). Frzb, a secreted Wnt antagonist, decreases growth and invasiveness of fibrosarcoma cells associated with inhibition of Met signaling.. Cancer Res.

[25] Nguyen DX, Chiang AC, Zhang XH, Kim JY, Kris MG (2009). WNT/TCF signaling through LEF1 and HOXB9 mediates lung adenocarcinoma metastasis.. Cell.

[26] Adams C, Totpal K, Lawrence D, Marsters S, Pitti R (2008). Structural and functional analysis of the interaction between the agonistic monoclonal antibody Apomab and the proapoptotic receptor DR5.. Cell Death Differ.

[27] Niida A, Hiroko T, Kasai M, Furukawa Y, Nakamura Y (2004). DKK1, a negative regulator of Wnt signaling, is a target of the beta-catenin/TCF pathway.. Oncogene.

[28] Glass DA, Bialek P, Ahn JD, Starbuck M, Patel MS (2005). Canonical Wnt signaling in differentiated osteoblasts controls osteoclast differentiation.. Dev Cell.

[29] Hsu H, Lacey DL, Dunstan CR, Solovyev I, Colombero A (1999). Tumor necrosis factor receptor family member RANK mediates osteoclast differentiation and activation induced by osteoprotegerin ligand.. Proc Natl Acad Sci U S A.

[30] Cho SJ, Vallès Y, Giani VC, Seaver EC, Weisblat DA (2010). Evolutionary dynamics of the Wnt gene family: a lophotrochozoan perspective.. Mol Biol Evol.

[31] Binnerts ME, Tomasevic N, Bright JM, Leung J, Ahn VE (2009). The first propeller domain of LRP6 regulates sensitivity to DKK1.. Mol Biol Cell.

[32] Yasui N, Mihara E, Nampo M, Tamura-Kawakami K, Unno H (2010). Detection of endogenous LRP6 expressed on human cells by monoclonal antibodies specific for the native conformation.. J Immunol Methods.

[33] Quarto N, Wan DC, Kwan MD, Panetta NJ, Li S (2009). Origin Matters: Differences in Embryonic Tissue Origin and Wnt Signaling Determine the Osteogenic Potential and Healing Capacity of Frontal and Parietal Calvarial Bones.. J Bone Miner Res.

[34] Zhou H, Mak W, Kalak R, Street J, Fong-Yee C (2009). Glucocorticoid-dependent Wnt signaling by mature osteoblasts is a key regulator of cranial skeletal development in mice.. Development.

[35] Zoltewicz JS, Ashique AM, Choe Y, Lee G, Taylor S (2009). Wnt signaling is regulated by endoplasmic reticulum retention.. PLoS One.

[36] Zhang Y, Appleton BA, Wiesmann C, Lau T, Costa M (2009). Inhibition of Wnt signaling by Dishevelled PDZ peptides.. Nat Chem Biol.

[37] Liu BY, Soloviev I, Chang P, Lee J, Huang X (2010). Stromal cell-derived factor-1/CXCL12 contributes to MMTV-Wnt1 tumor growth involving Gr1+CD11b+ cells.. PLoS One.

[38] Atwell S, Ridgway JB, Wells JA, Carter P (1997). Stable heterodimers from remodeling the domain interface of a homodimer using a phage display library.. J Mol Biol.

[39] Mohammad KS, Chirgwin JM, Guise TA (2008). Assessing new bone formation in neonatal calvarial organ cultures.. Methods Mol Biol.

[40] Khan Z, Vijayakumar S, de la Torre TV, Rotolo S, Bafico A (2007). Analysis of endogenous LRP6 function reveals a novel feedback mechanism by which Wnt negatively regulates its receptor.. Mol Cell Biol.

